# A rare neurological manifestation of a malnourished alcohol-dependent acute pancreatitis patient with Marchiafava–Bignami disease

**DOI:** 10.1093/gastro/goaa062

**Published:** 2020-10-01

**Authors:** Maciej Szmygin, Joanna Bielewicz, Krzysztof Pyra, Anna Szczepańska-Szerej, Konrad Rejdak

**Affiliations:** 1 Department of Interventional Radiology and Neuroradiology, Medical University of Lublin, Lublin, Poland; 2 Department of Neurology, Medical University of Lublin, Lublin, Poland

## Introduction

Marchiafava–Bignami disease (MBD) is a rare demyelinating disorder, usually associated with heavy chronic alcohol consumption. It may occasionally occur in patients who are not alcoholics but are chronically malnourished and patients with severe nutritional deficiencies [1]. White-matter degeneration affects the corpus callosum and results in neurocognitive impairment and dysfunction of the corticobulbar and pyramidal tracts [2]. Neuroimaging, diffusion-weighted magnetic resonance imaging (MRI) specifically plays a pivotal role in confirming the diagnosis of MBD and in determining prognosis [3]. The treatment remains controversial and shows variable results, with complete recovery being a rare outcome [4]. We present a case of an alcohol-dependent female patient with subacute MBD and concurrent acute pancreatitis.

## Clinical case report

**Figure 1.**
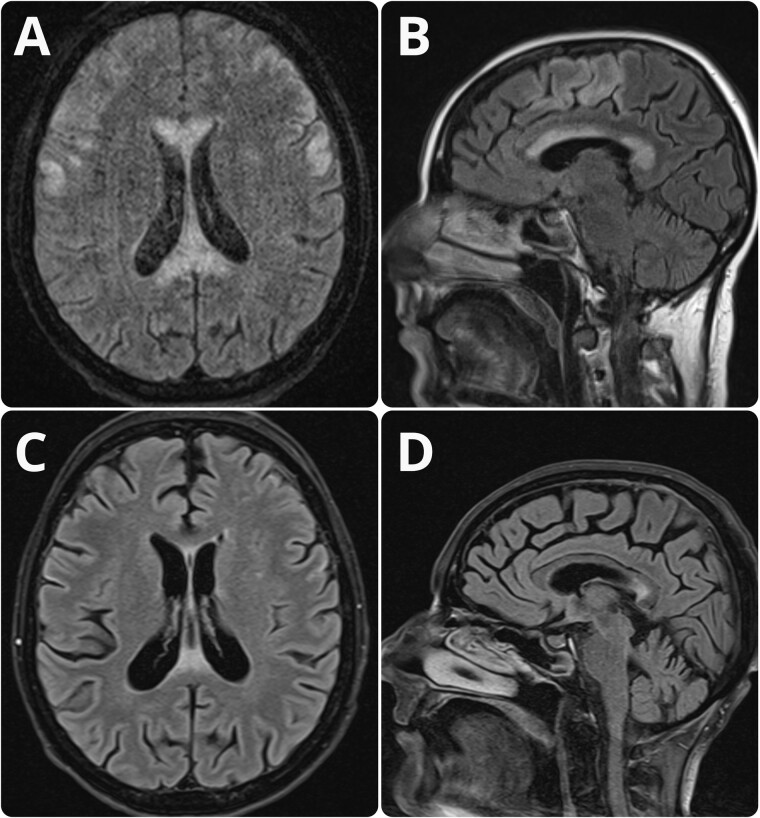
Significant decrease in the size of the primary area of involvement after 4 weeks of treatment compared with initial images. (A) and (B) T2-weighted MRI images showing the hyperintensity of the corpus callosum as well as the bilateral involvement of the cortical and subcortical regions of the frontal lobes. (C) and (D) T2-weighted MRI images revealing a significant decrease in the size of the primary area of involvement with residual hyperintensity of the splenium of the corpus callosum and complete regression of cortical and subcortical changes.

A severely malnourished 49-year-old woman (body mass index, 16.5) with a long history of alcohol dependency was admitted to the Emergency Department with speech impairment and gait abnormality that had developed gradually over the preceding 2 weeks. Neurological examination revealed cognitive impairment, dysarthria, and tetraparesis (more pronounced in the lower limbs), which indicated cortical, bilateral corticobulbar and pyramidal tracts impairment. Physical examination showed a distended abdomen with rebound tenderness. Blood tests revealed moderately elevated serum aspartate transaminase (90 U/L), alanine transaminase (72 U/L), gamma-glutamyltransferase (96.00 U/L), significantly elevated pancreatic lipase (324 U/L), and high C-reactive protein (143 mg/L). Additional laboratory tests detected a decreased level of total albumin (44 g/L), albumin (23.8 g/L), iron (0.31 mg/L), and calcium (0.078 g/L), but not vitamins B1, B6, B12, or other electrolytes. The lipid profile was within normal limits. Abdominal computed tomography (CT) showed a lack of pancreatic parenchymal enhancement with multiple pancreatic cysts suggestive of acute pancreatitis and significant liver fatty change. Diffuse hypodensity of corpus callosum was seen on cranial CT imaging, confirmed by hyperintensity of the corpus callosum on FLAIR and T2 images in MRI (Figure 1A and B). Similar hypodense changes were found in the cortical and subcortical regions of the frontal lobes. No corresponding hypointensity was observed on ADC images. MBD was diagnosed based on clinical presentation, history of chronic alcohol abuse, and characteristic MRI findings. The patient commenced pharmacological treatment consisting of calcium, iron, vitamin B complex, and a 5-day course of intravenous steroids with 500 mg methylprednisolone per day and dietary supplementation via percutaneous endoscopic gastrostomy.

After 4 weeks of treatment, the acute pancreatitis subsided, with pancreatitis-function biochemical markers normalizing (lipase 41.00 U/L, gamma-glutamyltransferase 56.00 U/L, C-reactive protein 17 mg/L). Follow-up MRI revealed a significant regression of abnormalities originally seen in the primary area of involvement with residual hyperintensity of the splenium of the corpus callosum on FLAIR and T2 images (Figure 1C and D). No significant improvement in the neurological and nutritional states was observed. Despite treatment and rehabilitation, the patient’s condition remained poor.

## Discussion

MBD occurs in patients with chronic alcoholism and, occasionally, in < 10% of all MBD patients [5], in malnourished non-alcoholics. Although necrosis or demyelination of the corpus callosum is specific for MBD, a pathophysiological mechanism explaining its particular vulnerability remains uncertain. It is attributed to the toxic effects of alcohol, but this is unlikely in view of the prevalence of alcoholism and the rarity of corpus-callosum degeneration. A nutritional etiology has been considered, but a specific factor has not been identified [6], although vitamin B deficiency is often suggested [7]. We did not find any specific nutritional deficiencies in our patient. Nonetheless, chronic malnutrition, whether caused by inadequate intake or gastrointestinal malabsorption, is detrimental and can impair the permeability of intracranial small vessels, which results in a poor nutritional supply to brain tissue.

Two subtypes of MBD are distinguished in clinical and neuroradiological classifications. Type A is characterized by an acute to subacute onset of altered level of consciousness and cognitive deterioration. The brain lesions depicted on MRI are localized in the entire corpus callosum. Extracallosal lesions are frequently found and are associated with a poor prognosis. Type B with insidious clinical onset develops progressively. Patients demonstrate a normal or slightly impaired level of consciousness. MRI shows lesions partially involving the corpus callosum and less frequently extracallosal. Type B is associated with a favorable prognosis.

Differential diagnosis includes other chronic, alcohol-related diseases of similar clinical presentation. Neuroimaging is crucial in achieving an accurate diagnosis and excluding other causes of neurological deficits. MRI, being of greater sensitivity, is the gold-standard diagnostic test, although bigger lesions are visible on CT. Typical callosal injuries are hypointense on T1 and hyperintense on T2 sequences (FLAIR and DWI), cause no mass effect, and may be visualized with contrast enhancement. Hyperintensities can also be found in other brain regions—cerebral lobes, hemispheric white matter, or basal ganglia (e.g. bilateral involvement of medial thalami, the mammillary bodies, and periaqueductal area are characteristic for Wernicke's encephalopathy). Central pontine myelinolysis shows lesions located in the central pons [8]. Conditions not related to alcohol abuse such as multiple sclerosis, acute disseminated encephalomyelitis, infarction, viral and bacterial infections, and HIV infection should also be considered in the differential diagnosis.

Effective treatment is not available due to the uncertainty of the etiology of MBD. Thiamine and other forms of vitamin B supplementation are routinely used. Haas *et al.* [9] reported a favorable response to corticosteroids, although other authors do not support these findings [10]. Regardless of treatment, partial or complete recovery is rare and most patients remain with severe neurological deficits.

## Conclusions

Our case report documents the diagnostic process of rapidly developing MBD in an alcohol-dependent and severely malnourished patient with concurrent acute pancreatitis. Despite treatment, only partial symptom resolution was observed, probably due to deep and irreversible cellular changes in the brain tissue and the gastrointestinal tract. However, prompt diagnosis and supportive treatment possibly prevented a dramatic or even fatal outcome. This case should raise awareness of the coexistence of different multiorgan complications and the need for a multidisciplinary approach. Alcohol-dependent and malnourished patients presenting with acute neurological deficits should be thoroughly investigated to exclude potentially fatal conditions such as MBD. The mutual interplay between the gastrointestinal tract and nervous systems has been a matter of great interest in recent years. 

## Authors’ contributions

M.S., J.B., K.P., and A.S.S. conceived and designed the project. M.S., A.S.S., and J.B. collected the data. M.S., J.B., A.S.S., K.P., and K.R. analysed and interpreted the data. M.S. and J.B. drafted the manuscript. All authors read and approved the final manuscript.

## Funding

None declared. 
